# Endotoxins and Biological Implications—Review

**DOI:** 10.1002/puh2.70397

**Published:** 2026-10-05

**Authors:** Uzair Ishtiaq, Nicolas Joly, Vincent Lequart, Patrick Martin, Marielba Morillo, Janne Rojas

**Affiliations:** ^1^ Department of Research and Development Paktex Industries Kamoke Pakistan; ^2^ Department of Life Sciences University of Management and Technology Lahore Pakistan; ^3^ ULR7519‐Unité Transformations & Agroressources Université d'Artois, UniLaSalle Béthune France; ^4^ Ecology and Nutrition Research Group Faculty of Pharmacy and Bioanalysis University of Los Andes Mérida Venezuela; ^5^ Organic Biomolecular Research Group Research Institute Faculty of Pharmacy and Bioanalysis University of Los Andes Mérida Venezuela

**Keywords:** endotoxemia, endotoxic shock, endotoxin, Gram‐negative bacteria, KDO, lipid A, lipopolysaccharide, *O*‐antigen, sepsis

## Abstract

Endotoxins, also known as lipopolysaccharides (LPSs), are structural components of the outer membrane of Gram‐negative bacteria and some cyanobacteria. They are among the most potent pyrogens and remain a persistent challenge in environmental, occupational, and clinical settings. This review brings together current understanding of their structure, stability, and methods of detection, while also highlighting the main routes through which humans are exposed. Evidence shows that inhalation of endotoxins, particularly in wastewater treatment plants, can impair lung function and trigger strong inflammatory responses, effects that may be intensified when combined with other agents such as glyphosate. Animal studies demonstrate damage to the gastrointestinal tract and blood exposure has been linked to wider systemic conditions, including obesity, insulin resistance, atherosclerosis, and metabolic syndrome. Endotoxins in water and wastewater further illustrate their public health relevance, as conventional treatment processes often fail to completely eliminate them. In biomedical contexts, residual endotoxins not only compromise device sterility but can also interfere with the accuracy of biomarker measurements in blood samples. Taken together, these findings emphasize the resilience of endotoxins under harsh conditions and point to the urgent need for more effective detection, sterilization, and monitoring strategies to protect both human health and the reliability of medical research.

## Graphical abstract

Graphical overview of endotoxin sources, exposure routes, biological effects, detection methods, and routes of elimination.

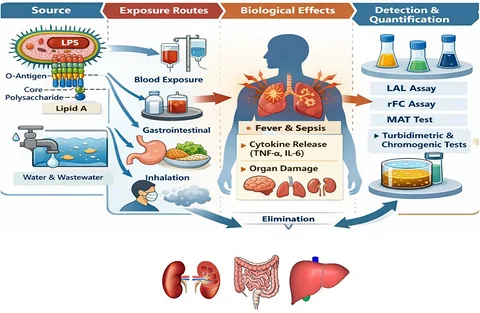

## 1 Introduction

Endotoxins, also referred to as pyrogens, are lipopolysaccharide (LPS) molecules located in the outer membrane of Gram‐negative bacteria [1]. The term “endotoxin” originates from the Greek words endon (inside) and toxicon (poison) [2]. The concept was introduced in the 1890s by the German bacteriologist Richard F. J. Pfeiffer, who distinguished them from exotoxins, soluble toxins secreted into the environment. In contrast, endotoxins remain bound within the bacterial cell wall and are released primarily upon bacterial lysis [3]. Figure 1 illustrates the general structure of the Gram‐negative bacterial cell wall [4].

**FIGURE 1:**
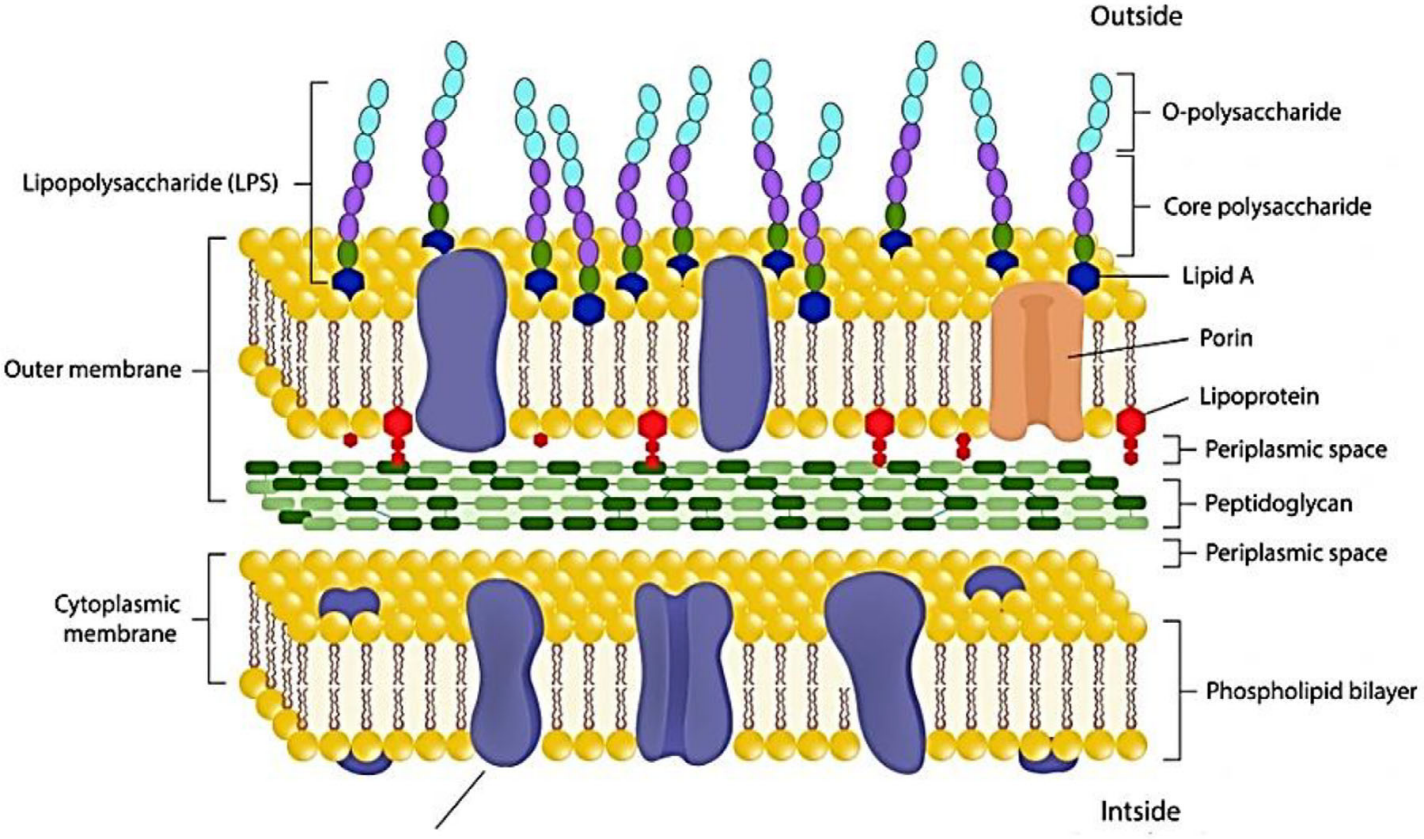
Cell wall of a Gram‐negative bacterium.

As shown in Figure 1, endotoxins are LPSs anchored in the outer layer of the bacterial outer membrane. Since the early 20th century, bacterial toxicity has been linked to lipid and carbohydrate fractions, which led to the adoption of the term “lipopolysaccharide.” When bacteria die and their cell walls are disrupted, LPS is released into the environment, where it becomes biologically active. Once in circulation, endotoxins stimulate the immune system and can trigger endotoxin shock, a condition associated with strong pyrogenic activity. Clinical manifestations include fever, hypotension, cardiovascular dysfunction, depressed cardiac contractility, septic shock, and related complications [1]. In severe infections, uncontrolled LPS activity can cause irreversible organ damage. Multiple occurrences of endotoxic shock may progress to coma and even death. The presence of endotoxins in the bloodstream is known as **endotoxemia** [5].

In comparison, the ingestion of endotoxins is generally not harmful, despite the abundance of Gram‐negative bacteria in the colon. Under normal conditions, endotoxins do not cause systemic effects because bacterial lysis does not occur on a large scale and the intestinal barrier prevents the exposure of the immune system to free endotoxin [6]. A major concern is the high stability of endotoxins. They are extremely resistant to heat and cannot be destroyed by standard sterilization methods. Effective inactivation requires exposure to temperatures above 180°C (at 5‐bar pressure) for at least 30 min [7]. This remarkable resistance highlights the importance of accurate detection and quantification methods to control their presence in medical, industrial, and environmental settings.

### 1.1 Structure, Physicochemical Properties, and Detection Assay of Endotoxin

As described previously, endotoxins are LPSs and represent the main component of the outer membrane of Gram‐negative bacteria. Since the 1980s, the structure of LPS has been elucidated, and it is now known that all endotoxins share a common structural pattern, which usually consists of three major regions (Figure 2):

- **Lipid A**—the proximal lipid region of LPS
- **The core**—a central region bridging the two parts
- **The *O*‐antigen**—the distal polysaccharide chain of LPS

**FIGURE 2:**
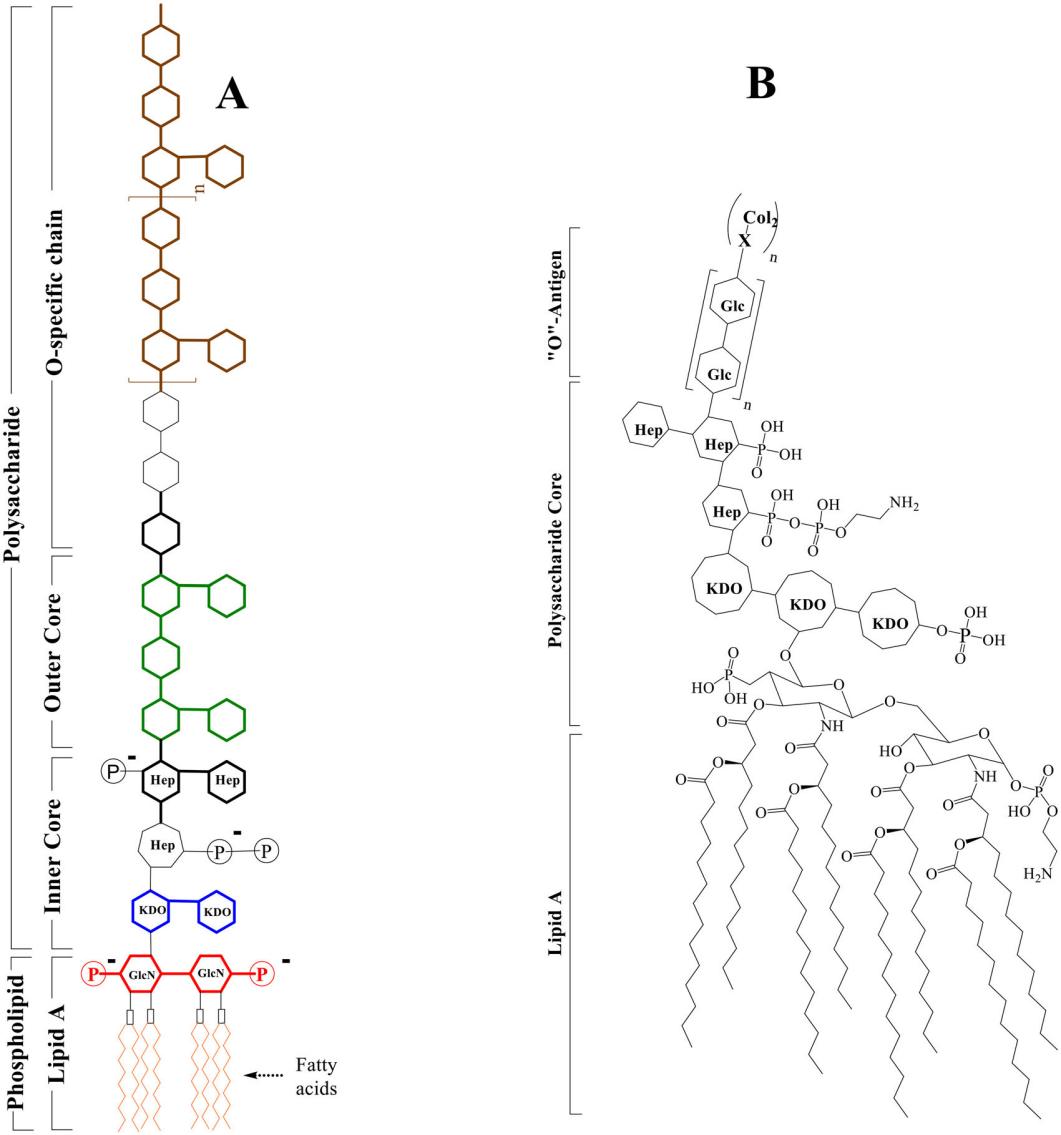
(A) General structure of bacterial endotoxins. (B) Representative structure of *Escherichia coli* LPS. LPS, lipopolysaccharides.

Lipid A, embedded in the lipid bilayer, is hydrophobic and represents the most conserved part of the LPS. The polysaccharide *O*‐antigen is hydrophilic and represents the most variable region, serving as the interface between the bacterium and its external environment. The core oligosaccharide is divided into two regions: an inner core, which is relatively hydrophobic, and an outer core, which is more hydrophilic [8]. Endotoxins are heat‐stable and resistant to both acidic and alkaline conditions. Their biological activity and ability to induce inflammatory responses remain intact even under extreme environmental stress such as fluctuations in temperature and pH. No structural damage occurs to the molecule, even during distillation or standard sterilization processes.

Since the late 1980s, the quantification of endotoxins in terms of mass units (e.g., ng/mL) has been replaced by the use of endotoxin units (EU/mL) [9]. Approximately, 1 ng/mL corresponds to 5–10 EU/mL [10, 11]. Figure 2 shows a general representation of an LPS from the Enterobacteriaceae family (*Escherichia coli*) [12].

The average molecular weight of an endotoxin monomer is about 10–20 kDa, with the lipid A region alone contributing approximately 2–4 kDa (p*K*1 = 1.3, p*K*2 = 8.2). Endotoxin monomers can self‐assemble into larger, stable aggregates due to their amphiphilic nature, which combines both hydrophobic and hydrophilic groups. These aggregates display considerable polymorphism, forming micelles of 300–1000 kDa and vesicles exceeding 1000 kDa [2]. According to [13], the size of an endotoxin monomer ranges from 0.002 to 0.02 µm, whereas self‐assembled vesicles range from 0.1 to 1.0 µm.

#### 1.1.1 Lipid A

The structure of lipid A is highly conserved among Gram‐negative bacteria, and within the same family it is almost identical. Lipid A consists of a β‐(1→6)‐linked diglucosamine backbone with attached fatty acid esters on both sugar units. The length and number of acyl chains vary depending on the bacterial species. Importantly, lipid A is the component responsible for the toxic activity of endotoxins.

#### 1.1.2 The “Core”

Generally speaking, the inner core of LPS is covalently linked to lipid A by the intermediate of an acidic sugar: KDO. The nucleus is made up of two main chains. The first chain is composed of two KDO sugars, and the second of three heptuloses, such as l‐glycero‐α‐d‐manno‐heptopyranose. The glycan residues of the inner core are generally phosphorylated or modified with groups containing phosphate; these help to stabilize the structure. As for the outer core of the LPS, it has a general structure that is more variable than the inner core. It is made up of more common hexoses like glucose, galactose, and *N*‐acetylglucosamine. The outer nucleus is also referred to as the “region of hexose.”

#### 1.1.3 The “*O*‐Antigen”

The *O*‐antigen consists of repeating oligosaccharide units, each formed by one to eight sugars, and can repeat 4–50 times. It may be a homopolymer (one type of sugar) or a heteropolymer (several sugar types). The *O*‐antigen is the most variable part of LPS and provides strain‐specific antigenic diversity. For example, more than 170 *O*‐serogroups have been identified in *E. coli* and 46 *O*‐serogroups in *Salmonella enterica*. LPS containing all three components, lipid A, the core, and the *O*‐antigen, is termed **smooth LPS**, whereas LPS lacking the *O*‐antigen is termed **rough LPS**. Importantly, lipid A is indispensable for bacterial survival; without it, Gram‐negative bacteria cannot maintain the integrity of their outer membrane.

In the 1950s and 1960s, German researchers Otto Lüderitz and Otto Westphal helped elucidate the structure of LPS. By subjecting LPS to acid hydrolysis, they disrupted the covalent linkage between lipid A and the core. This led to the discovery that lipid A, rather than the polysaccharide portion, is responsible for the endotoxin activity [6]. Because of its amphiphilic nature, LPS is difficult to extract in complete form. In hydrophobic environments such as oils, specialized methods are required to transfer LPS into an aqueous phase.

### 1.2 Biological Impact of Endotoxins in the Body

It has been previously explained that endotoxins can exert harmful effects on human health, primarily due to the lipid A component of LPS, which is toxic even at picomolar concentrations [6]. To illustrate their impact on the human immune system, Figure 3 presents a schematic representation of endotoxin activity and distribution in the body.

**FIGURE 3:**
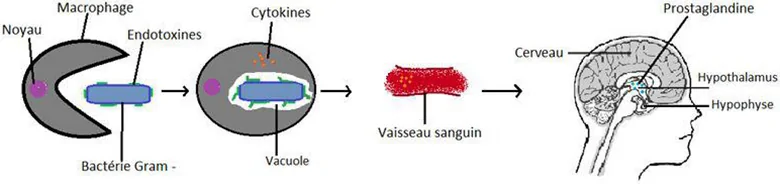
Schematic representation of endotoxin circulation in the body.

When bacteria enter the human body, they are rapidly engulfed into macrophage vacuoles, where degradation leads to the release of endotoxins. This process involves the LPS‐binding protein (LBP), which binds to the lipid A portion of LPS. The LPS–LBP complex then interacts with a specific receptor on macrophages. To date, Toll‐like receptor 4 (TLR4) has been identified as the primary receptor for LPS, recognizing it only in its non‐aggregated form [14]. The release of endotoxins activates macrophages to produce pro‐inflammatory cytokines, including interleukins (IL‐1, IL‐6, IL‐8), tumor necrosis factor‐α (TNF‐α), and platelet‐activating factor (PAF), which play a role in controlling endotoxin activity [6]. Once secreted into the bloodstream, these cytokines reach the hypothalamus in the brain. There, binding to specific receptors stimulates the production of prostaglandins, ultimately leading to an increase in body temperature.

## 2 Endotoxin Tests

### 2.1 History

The first agents causing fever, that is, “pyrogens,” were identified in 1912 by two bacteriological researchers: Hort and Penfold. They designed a pyrogen test based on injection of a sample containing endotoxins in rabbits [15]. Following this discovery, a large number of pyrogenic tests on rabbits continued to exist despite the variable sensitivity of this system. This made the development of alternative tests very necessary.

In 1956, a scientist, named Dr. Bang, conducted research on animal ecology studying horseshoe crabs and discovered a disturbance of blood clotting in those. Later on, he could recognize that the animal's blood cells (amebocytes) contain granules with an important clotting factor called “coagulogen.” This property gives the animal protection against bacterial endotoxins by creating a semi‐solid mass in his circulatory system [16]. In 1977, after numerous studies following Dr. Bang's discovery, the FDA3 of the United States approved the Limulus Amebocyte Lysate‐4 (LAL4) test to investigate drugs and other products by placing in contact with the blood of the horseshoe crab. This innovation made it possible to slow down and then eliminate the rabbit test, which was much slower, more expensive, and above all malicious towards animals. Nowadays, a new test on the search for endotoxins has been identified: the Monocyte Activation Test‐5 (MAT5). It uses human blood monocytes in vitro to detect pyrogens. This test produces results that are both reliable and reproducible. It was added to the PE in 2010 and to the USP in 2012 [17].

### 2.2 Test Details

#### 2.2.1 Rabbit Test

As described before, injecting the sample into rabbits carried out the early detection of endotoxins. Indeed, these have a similar tolerance to endotoxins to that of humans and thus constituted an ideal choice [18]. The purpose of the test was to observe their body temperature under regulated conditions. A substance was defined as containing pyrogens if it produced in the rabbit an increase in the body temperature higher than the normal limit.

However, this method was expensive, time‐consuming and created protests from animal rights activists. But the main drawback of this test was its inability to quantify the level of endotoxin in a sample.

#### 2.2.2 LAL Test

At the present date, the LAL test is mostly used because animal testing is no longer in use and has been prohibited. LAL is an aqueous extract of blood cells from the limulus or commonly known as the Atlantic “horseshoe crab.” The LAL test is based on the coagulation of the lysate of amebocytes contained in the blood with the presence of endotoxins: ALL reacts with bacterial LPS. This reaction is the basis of the LAL test, which is widely used for the detection and quantification of bacterial endotoxins [1]. This test is based on Dr. Bang's observation: Horseshoe crab blood clots when it is exposed to endotoxins. Thus, the amebocyte extract containing limulus blood is mixed with a sample suspected of endotoxin contamination. A reaction is observed if endotoxins are present.

Currently, to collect the blood from horseshoe crab, fishermen catch the animal and transport it to a blood collection center. Then the crabs are released into the wild. A marking on the animal is carried out before it is released to avoid taking blood from the same animal twice; this fishing takes place generally on the coasts of North America, Central America, and the Indian coasts. The animal lives in the wild on sandy bottoms. As for the blood cells, these are separated from the serum by centrifugation and then placed into distilled water. Then the cells start to swell and burst. This releases the delete products and leaves only chemicals inside the cell (the “lysate”), which are then purified and lyophilized. This process creates a LAL reagent, which will have coagulation property [19].

The LAL test remains better than the rabbit test in terms of animal abuse. However, it does not prevent that the makers of LAL have measured a mortality rate of nearly 30% in crabs bleeding. Thus, since 2003, a synthetic substitute for the LAL test has been available as EndoLISA. It is based on the Limulus C coagulation factor protein, produced by intestinal cells of genetically modified insects. This was considered more ethical and moral towards the protected species. However, the adoption of this test has been slow until 2016 when the European Pharmacopoeia classifies this bacterial toxin test as accepted. In fact, it was not as sensitive as LAL, but it remains effective in detecting and quantifying endotoxins [20].

#### 2.2.3 Monocyte Activation Test

The MAT uses human blood monocytes in vitro to detect pyrogens and helps to reduce the need for animal experiments. MAT offers several advantages over animal testing: It is based on the human fever response, provides a more accurate prediction of pyrogenic activity, detects both endotoxin and non‐endotoxin pyrogens, and can be applied across a wide range of products. It is also more accurate, cost‐effective, and time‐efficient. However, this method is not yet widely adopted in endotoxin analysis and therefore remains underutilized [18].

### 2.3 Endotoxin Main Exposure Tract

Different endotoxin exposures were studied, including blood, gastrointestinal, inhalation, and water contamination.

#### 2.3.1 Skin Exposure

It is considered that exposure to endotoxins through skin leads towards atopic dermatitis (AD), which is associated with T_H_2 cytokines and IgE, resulting in skewing the immune response in T_H_2 direction in which AD is not fully elucidated [21]. It was reported that bacterial endotoxins can penetrate through skin by irradiated latex gloves when samples were taken and outline different concentrations of endotoxin that remain present in the gloves; thus, it is noted that irradiation does not have any effect on endotoxins [22]. It was also assessed that domestic endotoxin from floor and bedding reservoir is an important source of HDM and cat allergens that enhance T_H_1‐type immunity and protect against allergen sensitization, whereas winter clothes are an important source of endotoxins, and recently washed clothes may have less toxin as compared to unwashed ones [23]. It is noted that the use of non‐sterile natural biomaterial wound dressings, such as calcium alginate, when tested with LAL, a high amount of endotoxin was detected in extract, confirms that pyrogens are endotoxins. The use of this type of wound dressing could also lead towards an exposure of endotoxin through skin abrasions [24]. It was confirmed that endotoxins are present on natural rubber latex products such as surgical gloves or Foley catheters that are being sterilized by gamma‐irradiation; when tested by LAL, they still confirm the presence of endotoxins that might pass through skin [25].

Figure 4 demonstrates that LPS enters the body through an open wound and binds to specific immune cells, such as TLR‐4 on monocyte, which leads to the activation of monocytes into macrophages and triggers inflammatory mediators such as cytokines, chemokines, and reactive oxygen species. On the other side, inflammatory mediators reach the wound site, such as neutrophil, which helps release ROS, leading to phagocytosis.

**FIGURE 4:**
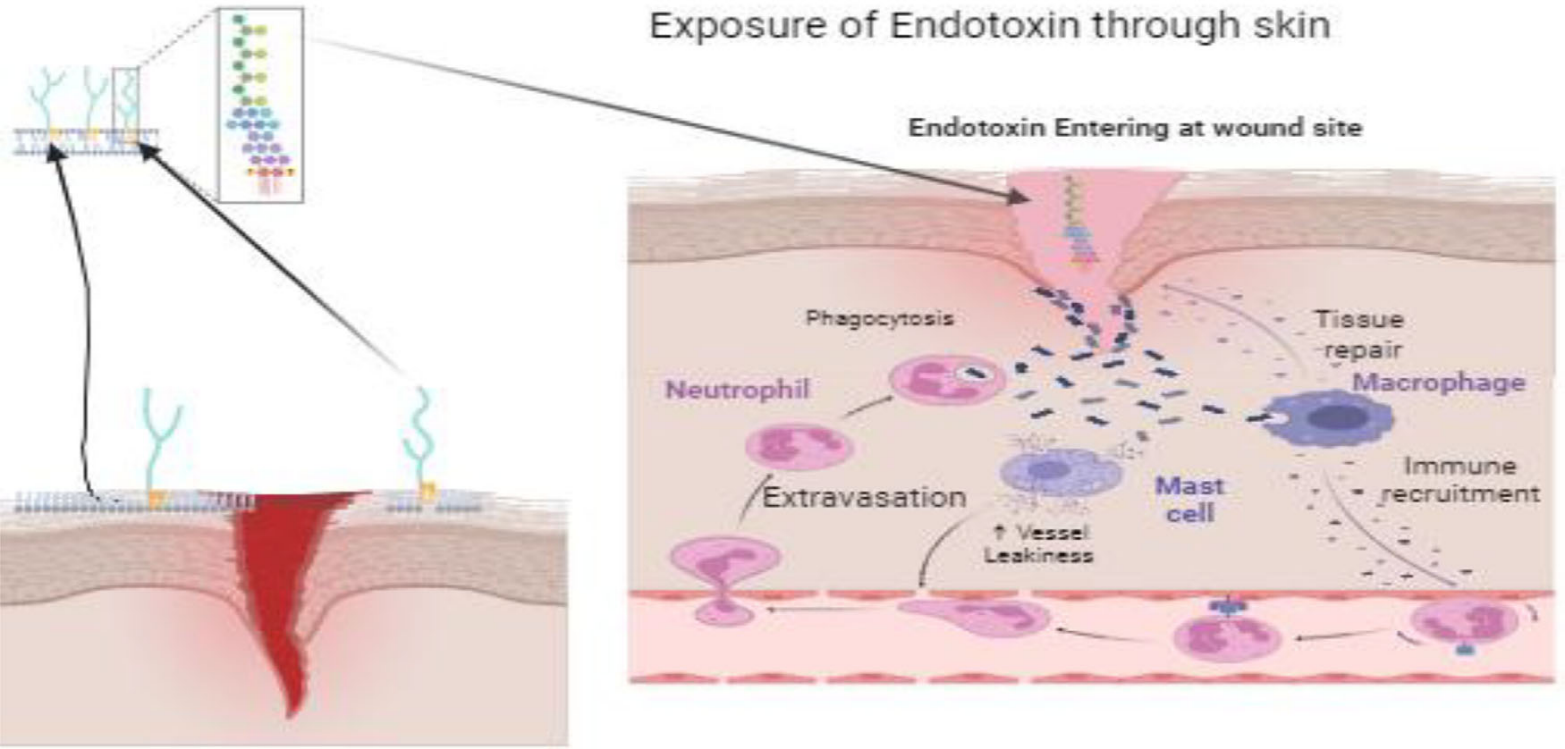
Entry of LPS into the human body through skin wounds.

#### 2.3.2 Blood Exposure

Endotoxins are bacterial LPS complexes residing in the outer membrane of bacteria [26]. It is widely accepted that in normal healthy human beings, a huge number of blood cells in bone marrow are produced on a daily basis, but this rate is accurately controlled according to physiological demands. Leukocyte production increases markedly during systemic infections.

LPS usually enters the tissues through blood circulation after several minutes of IV injection and keeps toxicity for a prolonged time [27]. Authors have found that LPS entry through IV infusion can prolong thrombin generation and partial activation time and reduce platelet number. To study hematological changes, including inflammatory responses, researchers often use LPS, the bacterial cell wall component sometimes found in contaminated infusions—as an experimental tool [28, 29, 30]. Copeland et al. [31] extensively studied inflammatory responses in mice and humans in response to LPS injection and observed similarities and dissimilarities. They injected the same stereotype of LPS *E. coli* type O113 in the same way into healthy male and female humans and male mice to determine the kinetics of inflammatory response. They injected the same LPS serotype (*E. coli* O113) in an identical manner into healthy male and female humans and male mice to determine the kinetics of the inflammatory response. Different parameters, such as temperature, blood pressure, complete cell count, and cytokine production, were determined. It was noted that 500 ng/kg of endotoxin in mice and 2 ng/kg in humans produced IL‐6 levels of approximately 1000 pg/mL. Lymphocyte and neutrophil responses differed between humans and mice, as observed in hematological analysis.

At the beginning, there was a dramatic increase in lymphocytes at 4 h after infusion, which then gradually returned to normal values. It showed a rapid initial increase, returning to normal levels after 24 h. Overall, there was a slight increase in leukocyte numbers in humans after 6–9 h of exposure, whereas no alterations were observed in mice (Figure 5). The blood profile in humans was reported to show leukopenia followed by leukocytosis, with significant changes in neutrophil counts [32, 33, 34].

**FIGURE 5:**
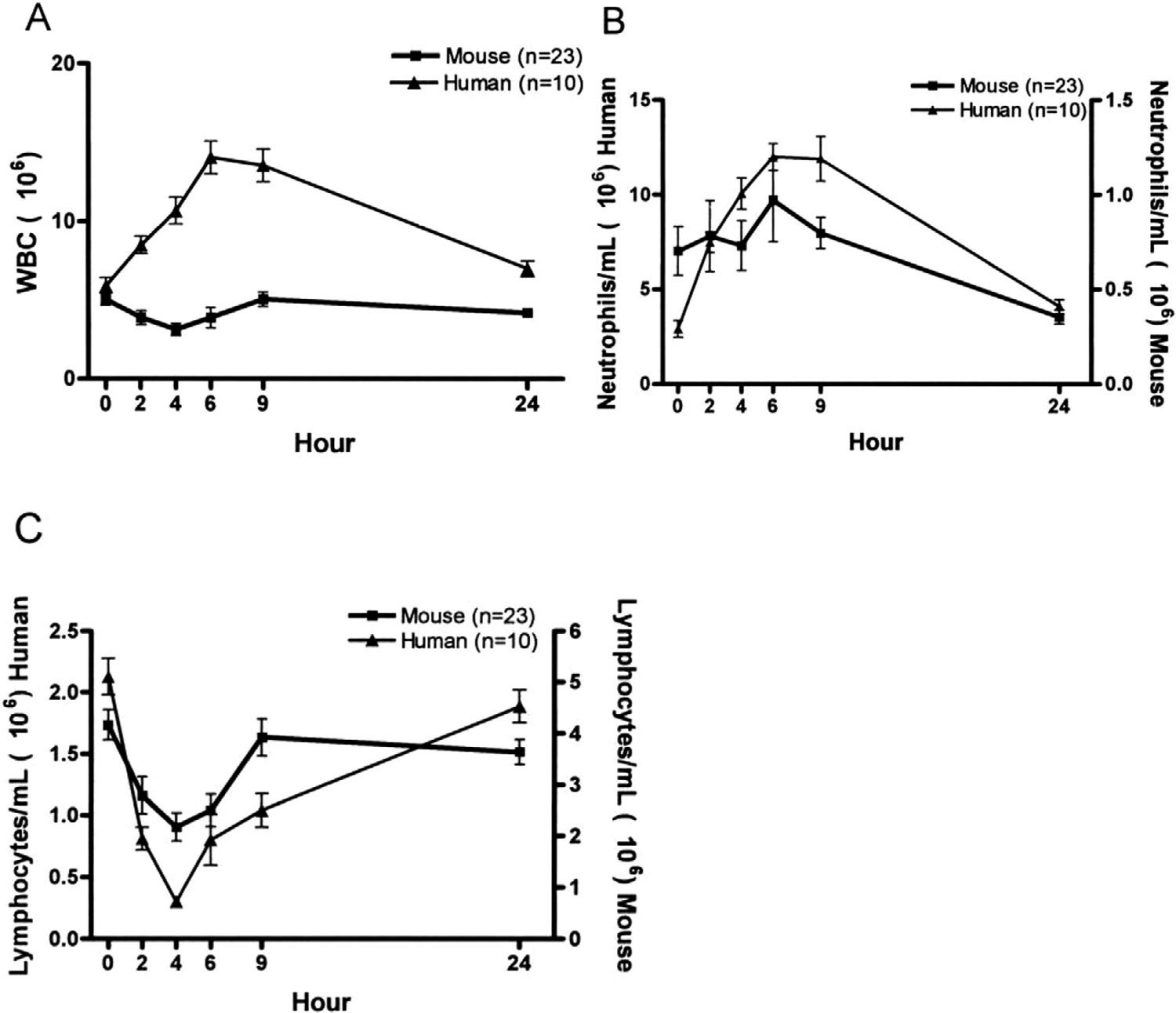
Hematological alterations after endotoxin infusion.

The exact pathogenic pathway of endotoxins is still not fully clarified, but several biochemical mechanisms have been identified. LPS can activate protein kinases and phospholipases (PLA2, PLD, PLC), altering monocyte membrane phospholipids, increasing fluidity, and stimulating the arachidonic acid pathway [35, 36, 37, 38]. This activation contributes to hypercoagulation, which can be blocked by PLA2 inhibitors [39]. Excessive PLA2 and PLC activity may destabilize cell membranes, leading to cell death and production of ROS [40, 41, 42, 43, 44, 45]. Downstream signaling includes NADPH oxidase activation with arachidonic acid release and PLC‐induced activation of PKC, which stimulates inositol‐3,4,5‐triphosphate and diacylglycerol (DAG) production [46, 47, 48, 49]. Stief reported that LPS‐induced coagulation is a key pathological feature [50]. In septic shock, PLA2 levels increase up to 500‐fold, with activity rising 200‐fold [51]. In healthy individuals, intravenous LPS infusion increased PLA2 activity 10‐fold [52]. Furthermore, mice with fewer PLA2 receptors were resistant to endotoxin shock, confirming its pathological role [53].

At the genetic and hematological level, intravenous LPS alters gene expression related to signaling, immune defense, and recognition pathways. T‐lymphocyte–associated genes are suppressed, whereas receptors, such as cluster differentiation‐14 (CD14), FPRL1, TLR1, MARCO, complement receptors (C3a, C3b/4b, C1q), properdin factor P, bactericidal permeability‐increasing protein, and Fcγ receptors (LILRA3, LILRB2), are upregulated [54]. These changes are accompanied by monocytopenia, thrombocytopenia, and the release of negatively charged DNA and microparticles from lysed monocytes and endothelial cells [55, 56]. The lysis of ∼200 nucleated cells/µL can release about 3 µg/mL plasma DNA, further promoting coagulation. Ultimately, these processes contribute to disseminated intravascular coagulation (DIC) [57], with sepsis‐associated DIC considered pathophysiologically similar to coagulopathy caused by snake envenomation [58].

#### 2.3.3 Gastrointestinal Exposure

Endotoxins having hydrophilic polysaccharide chain and lipophilic tail can form micellar granules with bile acids in lumen of intestine. The diameter of such endotoxin aggregates is greater than 100–200 Å; hence, they cannot cross paracellular spaces [59]. Endotoxin entry into the cell is dependant on structure of plasma membrane, for example, lipid rafts and clathrin‐linked mechanisms [60]. Using enterocyte cell membrane or by receptor‐dependent transcytosis, endotoxin can directly be absorbed following exocytosis at the epithelial cell membrane [59, 61, 62, 63].

LBP and CD14 when associated, endotoxin penetrates through the cell membrane [64]. A mechanism that is newly discovered shows that endotoxins can penetrate into the goblet cell‐associated pathway that is also known as GAP pathway for the large molecules to the immune cells in the intestine and not into the colon [65]. Similar to the triglyceride chains, endotoxins can also be absorbed with same mechanism [66].

LPS exerts its deleterious effect mainly by triggering inflammation by TLR‐4 pathway [67], which has been considered one of the potent triggers of systemic inflammation and multiple organ failure in critical illness [68]. After TLR‐4 activation, LPS triggers inflammatory pathways that result in coagulopathy, endothelial, and cardiovascular dysfunction [69, 70].

With the help of PRRs (pattern recognition receptors) of the gut defense barrier, gut epithelia detect pathogenic microbes using TLRs that trigger proinflammatory pathways and alert the host to potential infection [71]. CD14, which is linked with TLR‐4 as a cofactor, can sense LPS and other components based at the gut [72, 73] and circulate in system with the help of paracellular transport when LPS interacts with other structures present on the epithelial cells [72]. This interaction leads towards the activation of NF‐κB (canonical nuclear factor kappa beta) and some cytokines, including IL‐1β, IL‐6, IL‐8, TNF‐α [74], increasing the intestine permeability [75]. Although for intestine barrier, IL‐10 acts as a protective agent [76], myosin light chain kinase and its activity can be increased with intestine permeability due to the LPS [77].

Through intestinal epithelium, LAP is moved into the lymph and triggers macrophages in mesenteric lymph node [66]. Part of endotoxin is crossed through the portal vein to the liver, where hepatocytes, Kupffer cells, and inflammatory cells scavenge its amount to enter the systemic circulation. Kupffer cells activate and release more proinflammatory cytokines in systemic circulation [78].

These cytokines activate the release of acute phase protein (APP) such as haptoglobin (Hp), c‐reactive protein, SAA (serum amyloid A), and LBP [79]. The latter performs multiple functions in neutralizing, removing, and mounting an immune response against endotoxin. A small amount of LPS enters the systemic circulation and binds to LDL and HDL (low‐density lipoprotein and high‐density lipoprotein, respectively), a process triggered by LBP and CD14 [80, 81]. Endotoxin attached with lipoproteins is removed either by liver hepatocytes or white adipose tissue to circumvent its deleterious effects on the host. Endotoxin stored in white adipose tissue is attacked by macrophages, leading to secrete more proinflammatory cytokines that induce inflammatory state in systemic circulation [82].

Figure 6 illustrates that LPS enter the body along with food and reach the stomach, where they adhere to the TLR‐4 receptor and then enter the stomach lining, where macrophages and other immune responses are activated to clear them and cause inflammation. If the stomach lining is damaged, these LPS will enter into the bloodstream by paracellular pathway, which is the space between cells in the epithelial layer, and move towards the liver through portal vein (as liver is the first organ that encounters the blood from GI tract). Once LPS enters, accumulates, and activates the Kupffer cells that engulf these LPS. In the case LPS amount is too high and Kupffer cells are not as per demand, these LPS enter into the circulation system and lead to inflammation of the whole body that is also known as endotoxemia.

**FIGURE 6:**
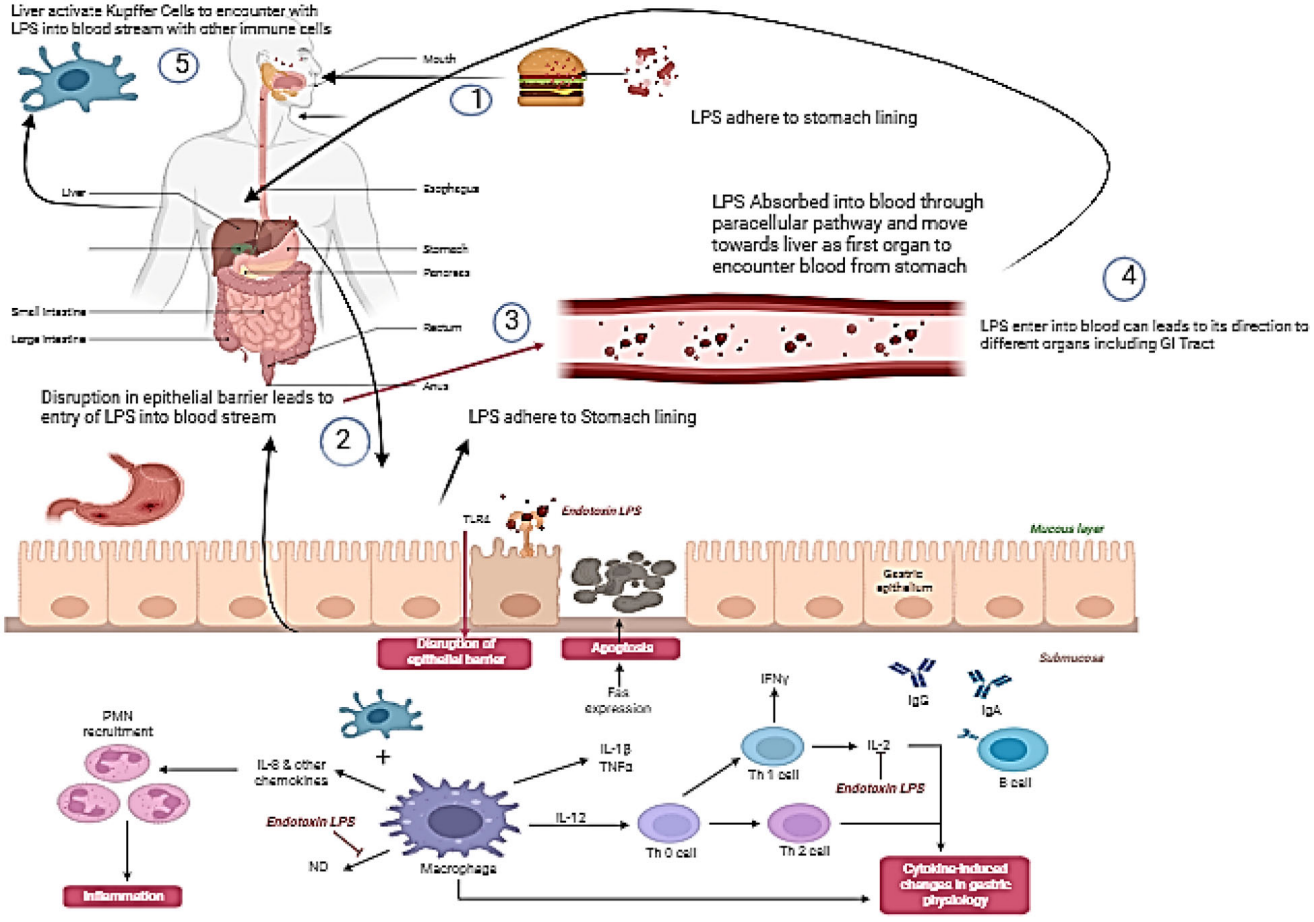
LPS enters the body through the GI tract.

#### 2.3.4 Inhalation Exposure

In lungs, mucociliary system is the first line of defense in the lungs that is located at nasopharynx performing the task to remove particulates that are dropped in the airways. Although bacteria and their by‐products might reach the alveolar membrane at the mucociliary system and be detected by the macrophages, which are sentinel cells of the innate immune system present in the lungs, this innate immune response constantly maintains vigilance for the microbes and their by‐products that reach the alveolar membrane [83]. At alveolar spaces and terminal airways, when endotoxin tries to enter, it faces a lipid‐rich environment where lipids, surfactant, and protein‐linked surfactant line the thin aqueous layer covering the whole epithelium. sCD14 and LBP are the main components of this alveolar fluid, and membrane CD14 triggers the macrophage present at the alveolar to the LPS in vitro [84]. The concentrations of sCD14 and LBP in bronchoalveolar fluid as well as in plasma increase more than 10 times in patients suffering from acute lung injury; the concentration of LBP and its source are still unknown in the injured lungs [85].

Proteins and lipids that can bind and sense the LPS and regulate its biological activity can also compromise the alveolar environment with the help of lipid A moiety of LPS, which reduces the biological activity of LPS in vitro and binds to SP‐A [86].

Comparing the injured lung with the healthy lungs, SP‐A concentration and surfactant lipid concentration are reduced [87, 88]. Further research is needed on the role of plasma lipoproteins in the lungs during the regulation of bioactivity of LPS. The role of pathways, such as LPS, LBP, or CD14 in the lungs, shows that LBP is an alveolar epithelial cell product that is mainly regulated by the proinflammatory cytokines. It may show therapeutic effect if this pathway may inhibit a rabbit treatment with a monoclonal antibody specific to antiCD14, which protects them from further encounters when injected even after the first LPS encounter [89].

In healthy and injured lungs, different biological activities were reported, including interaction with the inflammatory system function. During normal air space, when LPS tries to enter the system, it faces 250–500 µg/mL concentration of SP‐A that is very high in diluted bronchoalveolar lavage fluid [88]. Similar 50–100 ng/mL in undiluted alveolar fluid concentration of LBP was reported as very low concentration [85]. IL‐6, IL‐1β, and TNF‐α that are present in the air spaces, and the surfactant lipids concentration and SP‐A concentration were less than 10% of the normal ranges of 25 µg/mL; in patients with acute lung injury, similar concentrations of LBP are 5–10 µg/mL that is 100 times more than normal [87, 88, 90]. In air spaces, LPS biological effects increase in the lungs due to the dominance of the LBP, whereas these findings are very important to asthmatic patients who are exposed to inhaled allergens and LPS in the workspace, as well as to patients who are suffering from acute lung injury (Figure 7) [91].

**FIGURE 7:**
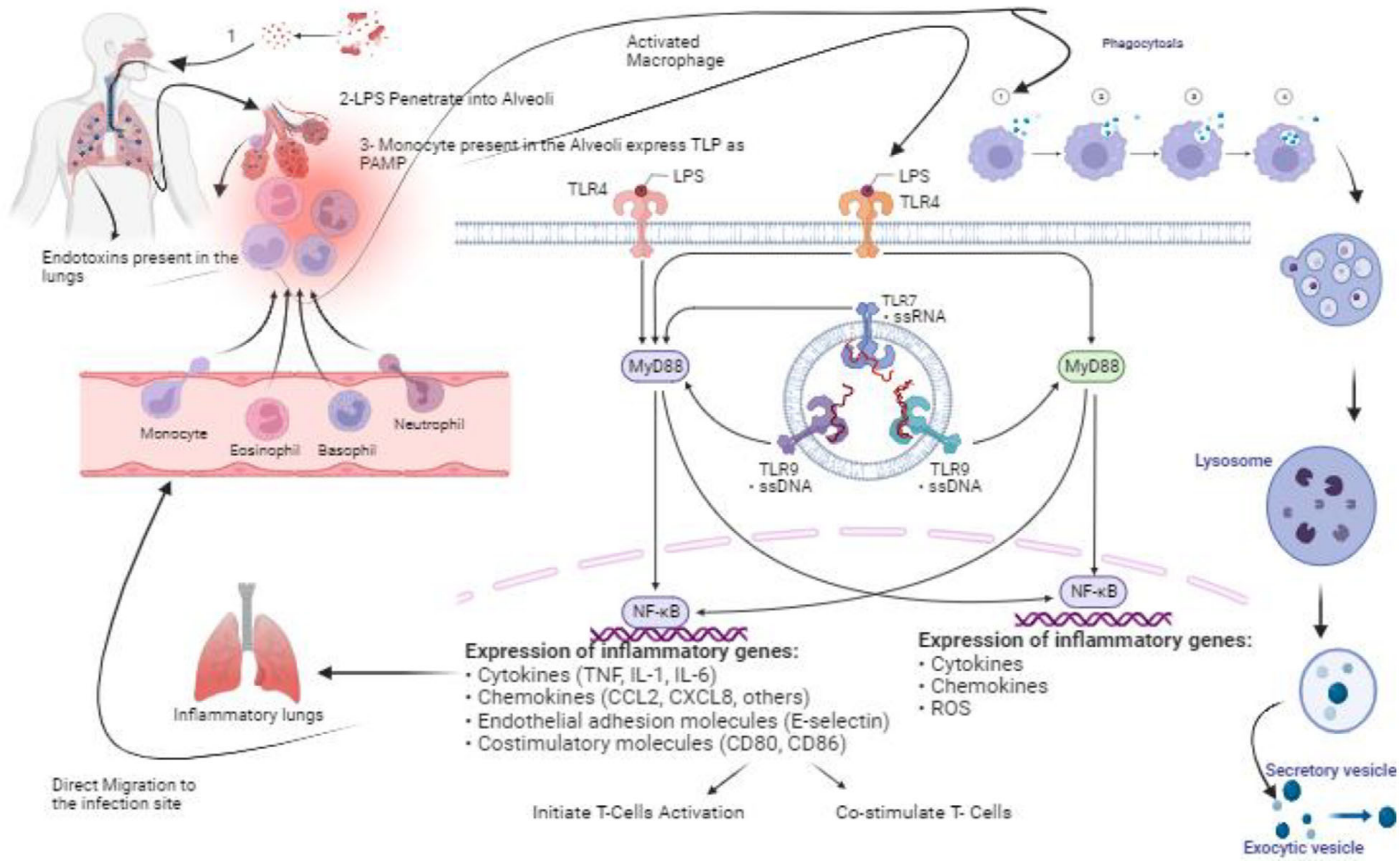
LPS enters the body using inhalation route. LPS, lipopolysaccharide.

For microbes, particulates, and environmental gases, there is a physiological barrier of epithelial cells and alveolar macrophages present in the airway compartment. During the serious injury to the lungs, it could lead to the life‐threatening condition as well as some serious illness, with a condition known as acute respiratory syndrome also known as ARDS. Once any foreign antigen or microbes try to enter, they have to deal with the alveolar macrophages as they are the first cells with which they have to interact, and these are located in the air tissues [92] and play an important role upon activation during any acute inflammatory injury to the lungs. Cytokines are released, such as TNF‐α and IL‐1β, as well as some chemokines, such as monocyte chemoattractant protein 1 also known as MCP‐1 and monocyte chemoattractant protein 1 beta that is also known as MCP‐1β, are due to these macrophages present in the lungs [93].

CD54 and ICAM‐1 as well as VCAM‐1 (intercellular adhesion molecule 1 and vascular cell adhesion molecule 1, respectively) are immunoglobulin superfamily adhesion molecules. For the migration of white blood cells to the inflammation site, ICAM‐1 and VCAM‐1 play a vital role [94] and attach effector cells to the respiratory epithelium [95]. When these mediators are together, they play a vital role in the orchestration of an inflammatory response, especially in neutrophil recruitment. Other studies have shown that these cytokines and chemokines not only originate from the alveolar macrophage but also from different epithelial cells [96].

LPS enters the body through contaminated air and starts to accumulate in the alveoli of the lungs. Alveoli macrophages start to engulf these LPS and trigger the activation of TLR‐4 pathway (Figure 7). This TLR‐4 leads towards the activation of NF‐κB and leads towards the production of immune responses such as cytokines, chemokines, and reactive oxygen species, and causes inflammation in the lungs. This also leads to the activation of neutrophils, macrophages, and dendritic cells that start phagocytosis process. This phagosome that contains LPS fused with lysosomes makes a phagolysosome. Phagolysosomes have some digestive enzymes that degrade LPS and release it into the extracellular space by secretory vesicle, whereas exocytic vesicles remove this waste by fusing with the plasma membrane.

#### 2.3.5 Water and Wastewater Exposure

Pathogens and biological agents could be removed from water, whereas toxins from these agents may be left behind. Very few reports are available on the harmful contents of these agents in water and other environments [97, 98, 99, 100]. In humans, these toxins remain, and some adverse effects are identified, such as endotoxins that produce toxic shocks and immune system dysfunction [100].

These endotoxins are reported to produce severe illness, including flu‐like, GI, airway, and neurological symptoms [101, 102]. This illness is due to tap and bath water that are contaminated and have been observed and not detected by routine medical diagnostics as well as bacteriological testing, as they have a short life span [103, 104]. It was reported that blooming water, sewage water, and air in the wastewater treatment plant have a high number of endotoxins [105, 106, 107]. During the encounter of LPS, different adverse effects, such as a high number of macrophages and neutrophils and certain chemokines and cytokines in the sputum and bronchoalveolar lavage fluid, are produced and might have fever‐like symptoms [107]. For the detection and measurement of LPS, different indicator microbes are used that produce signals on the presence of biological hazards, whereas these are reported in two categories of composting plants. One of these processes is for the industrial production of substrate to cultivate button mushrooms and later for the processing of biodegradable waste. Both have a high level of pathogens, including respiratory bioaerosols and endotoxins [108].

An investigation carried out with 48 workers involved in the collection and sorting of waste, whereas they recorded the highest exposure of the endotoxins to those workers who perform sorting waste, incineration, and recycling of these waste tasks. Out of these 48 workers, 35% were found to be exposed to 1000 EU/m^3^, whereas average exposure, which depends on humidity, region, and temperature, was 1123 EU/m^3^ [109]. Workers who are working at wastewater treatment plants are reported to a complex mixture of non‐infectious and some infectious microbes coming from products and some toxic gases. These workers experienced symptoms extending from the CNS to respiratory and GI tract [110, 111, 112, 113].

Illness was reported in workers who were performing wastewater treatment, with symptoms and lung infections, indicating that they have been exposed to a severe level of endotoxins [112, 113].

Some studies showed that there is a relation between exposure when handling sewage plant dust and specific inflammatory pneumoproteins and inflammatory biomarkers [114, 115]. Although H_2_S and LPS may have targeted organs such as respiratory system, exposure to this mixture of contaminants in low levels in the airway is less studied (Table 1) [116].

**TABLE 1:** Level of endotoxin contamination in water and wastewater.

Type of water and wastewater	Information on sample	Country/Region	Total endotoxin	Reference
Drinking water	Tap water	Manchester, UK	7000 EU/mL	[117]
Processed water	Lake Chivero	Zimbabwe	1000–7750 EU/mL	[118]
Freshwater	Containing cyanobacteria	Finland	38,000 EU/mL	[119]
Treated water	Yodo River	Japan	2430 EU/mL	[120]
Fresh water	River	The United States	1000 EU/mL	[121]
Stagnant water	University campus	China	>10 EU/mL	[122]
Aquaculture	Fish ponds			
Purified pharmaceutical matric	Clean water	Japan	0.5 IU/mL	[123]
Reclaimed water	Chlorinated	China	1584 EU/mL	[124]
Reclaimed water	Chlorinated	China	422 EU/mL	[125]

#### 2.3.6 Medical Devices Exposure

Sepsis is one of the most common issues faced by healthcare, with bloodstream catheter infection mainly caused by Gram‐negative bacteria; however, its propagation is still unclear. It was concluded that the use of wound dressing impregnated with chlorhexidine acetate, such as Sera‐Tulle, can control all bacterial growth around the needle or catheter. One of the most important aspects of endotoxin (LPS) exposure is medical devices such as Syringes, IV sets, IV cannulas, cord clamps, Gauze Swabs, HME Filters, Foley, ETT, urine bag, blood bag, and many more non‐sterilized medical devices or those are not properly sterilized and have some endotoxins within. To identify their presence, LAL test is widely used around the world. European Pharmacopeia and the US FDA specify endotoxin value limits of 0.006 EU/mL for devices that have contact with liquor; 0.5 EU/mL for medical devices; and 5 EU/kg/h for parenteral drugs [126, 127]. To remove these particles, autoclave process is not enough; dry heat at 180°C or chemical methods, such as EO or H_2_O_2_, are able to fully extinguish the endotoxins when treated for several hours [128]. Medical devices such as infusion sets (IV sets) may cause problem to the patients when particles are present in them, whereas FDA has set standard acceptance criteria of a minimum 90% recovery of 10–25 µm and a minimum recovery of 80% for ≥25‐µm particles [129]. For the analysis of particulate contamination, different transfusion and hemodialysis sets are being used where particle‐free water into these tubes was injected, and the particle contamination was measured at different times, whereas this injected water was passed through filters of different sizes, such as 0.8, 0.45, and 0.22 µm, and contamination was observed [130]. LPS and other exogenous pyrogen when enter into body subsequently shown to be IL‐1, and this IL‐1 is the cause of fever in human and animal body (Figure 8) [131]. It was investigated that the presence of endotoxins in plastic blood collection tubes significantly impacts biomarker assay measurement and leads towards inaccurate results and affects clinical decision‐making.

**FIGURE 8:**
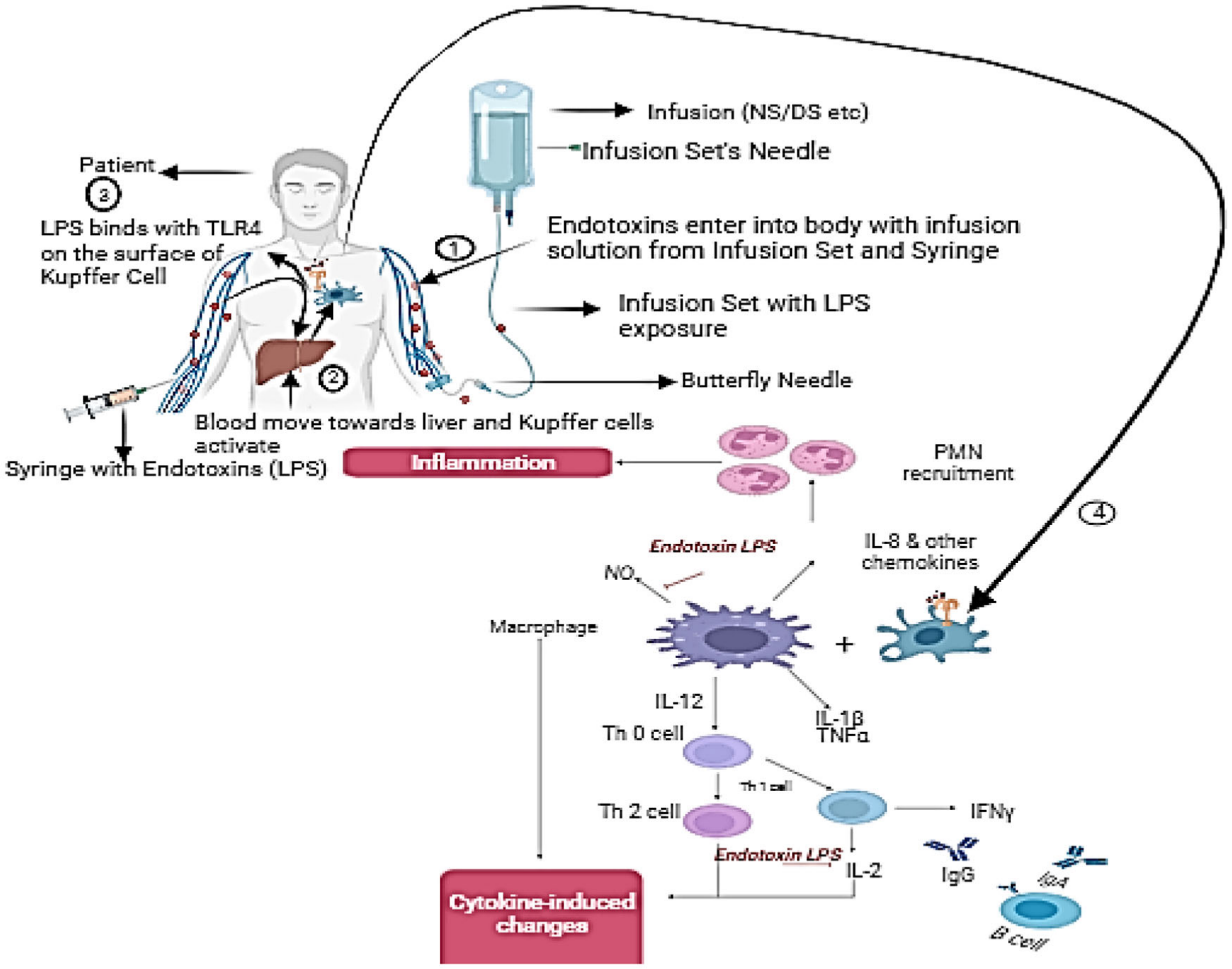
LPS enter into body by medical devices into bloodstream. LPS, lipopolysaccharide.

Endotoxins enter into body with the use of non‐sterile medical devices or compromised sterile medical devices. LPS enter the body and airways, eventually accumulating in the alveoli of the lungs. The alveolar macrophages, which are present there, begin to engulf these LPS and trigger the activation of a pathway called TLR 4. This activation of TLR 4 leads to the activation of NF‐κB and subsequently results in the production of responses such as cytokines, chemokines, and reactive oxygen species. This immune response causes inflammation in the lungs. Additionally, it also activates neutrophils, macrophages, and dendritic cells, which initiate a process called phagocytosis (Figure 8).

#### 2.3.7 Fish Industry

Farming exposure to endotoxins in seafood industry is relatively low. A Norwegian study reported the data on blood profiles, lung function measurements, and some questionnaires among 72 workers that mostly show symptoms of cough, chest tightness, and wheezing. Endotoxin levels were in the range of 0.30–29.0 EU/m^3^ [132]. Consumption of fish that have hazardous levels of histamine produces scombroid poisoning [133, 134, 135]. As fish decompose, histidine is transformed into histamine; this decarboxylation is mediated through a bacterium [136]. Although other biogenic amines, such as agmatine, tyramine, cadaverine, and putrescine, can be produced during the spoilage of the fish due to amino acid precursors of bacterial decarboxylation [137, 138, 139, 140], this toxicity is due to the inhibition of histamine‐metabolizing enzymes in the small intestine [141, 142, 143].

Detection of histamine and biogenic amines in some specific fish species has become important for food safety and human health. These biogenic amines are indicators for the seafood as quality assessment is limited [139]. An important role is played by the high temperature in which a factor contributes to histamine and amine production in most fish species [144, 145]. Improper handling and storage of fish meat at high temperature are the main reasons behind this [146, 147]. Fish species named Mediterranean parameter for quality control, such as sardine, mackerel, and hake, are not studied as these are found at cold water [148]. *Porphyro monasging val*, an important endotoxin named gingipains, was documented. It was noted the effects in a hyperglycemia‐induced zebrafish for the evaluation of oxidant/antioxidant status [149]. Systemic disorders are associated with periodontitis that affect a larger population, with diabetes mellitus and obesity, which are both linked with the hyperglycemia and systemic inflammation [150]. Diabetes is one of the most common and serious diseases, similar to periodontal diseases. These are characterized by hyperglycemia and low‐grade inflammation [151].

Body oxidative stress levels that even cause cell death and increase systemic inflammation are altered by the chronic hyperglycemia [152, 153]. During inflammation, APPs are changed [154]. Although c‐reactive protein and YKL‐40 are APPs and are linked to hyperglycemia‐induced diseases [155, 156, 157, 158], it was concluded that endotoxins of *Porphyromonas gingivalis* were used to treat hyperglycemia‐induced zebrafish when gingipain was administered. Results showed negative effects on diabetes. Oxidant–antioxidant compromised balance impaired glucose metabolism [149].

### 2.4 Route of Endotoxin Elimination

Even low concentrations of LPS are essential to remove from the body, as they produce inflammation stimulators, whereas new perspectives are offered during the detoxification of LPS. Multiple mechanisms are reported for the removal of LPS from the body, including molecules that bind to LPS and prevent their actions and inactivate them, which leads towards reticulo‐endothelial system where enzymes degrade LPS [159]. For the removal of endotoxins, main mechanism for the detoxification is a molecule that binds with the LPS and prevents it from engaging with TLR‐4, destroys its lipid A moiety, as well as decreases its activity, and leads towards liver and spleen for the adaptation that modifies targeted cell response to LPS. Term detoxification comprises reversible (sequestration, binding) and irreversible chemical modifications of LPS, as well as some indirect mechanisms that neutralize proinflammation host response to the LPS [160].

#### 2.4.1 Anti‐LPS Antibodies

In humans, endotoxins are neutralized by binding with antibodies such as IgG and IgM that make a complex and activate the classical complement pathways where RBC and macrophage receptor also bound and clear into the spleen, liver, and reticulo‐endothelial tissues [161, 162]. Antibodies that neutralize LPS bind with protein such as HDL and BPI (bactericidal permeability increasing protein) [163, 164].

#### 2.4.2 LPS Binding Proteins

LPS can also inactivate when protein binds to them, mannose‐binding lectin can stop LPS to interact with TLR‐4 or MD‐2 receptors by stimulating C3 and increasing clearance that is similar to IgM. Here, BPI is stored in neutrophil granules and released when cells undergo to the phagocytosis or exocytosis process.

BPI kills the bacteria by disrupting the cell wall and prevents the binding protein from transferring to CD14 that favors monocytes and neutrophils to avoid cell activation. Similar proteins such as azurocidin can also bind with LPS and augment LPS‐induced TNF‐α, IL‐8, and prostaglandin E2 production by monocytes [165]. LPSs that are circulating in the plasma are bound to some lipoproteins, and these lipoproteins inactivate them. A lipid moiety will not be exposed while transferring LPS to lipoprotein [166]. When LPS interacts with rBPI (21) and passes them to intermembrane space, it enables their interaction with the inner membrane and produces antimicrobial activity. These interactions produced within bloodstream an electrostatically with LPS clusters and neutralize its toxicity [167].

#### 2.4.3 Deacylation of LPS

LPSs have a highly conserved structural domain of lipid A that is identified by TLR‐4 receptor or MD‐2; upon full recognition, phosphate and acyloxyacyl linkages are degraded into inactive or partial agonist molecules [168, 169, 170].

#### 2.4.4 Dephosphorylation of LPS

LPS resides within the lipid A part that is vital for its biological activity and causes toxicity. Injecting placental alkaline phosphate stops mice from producing harsh effects of LPS. Refs. [171, 172] report that for the detoxification of LPS, extracellular and intracellular dephosphorylation is required.

#### 2.4.5 Alkaline Phosphatase

It was reported that the infusion of oral intestinal alkaline phosphatase (iAP) shows some good effects on the severe damage of intestinal epithelium where inflammation was produced, whereas endogenous iAP can counteract LPS disease effects [173].

#### 2.4.6 Liver and Reticulo‐Endothelial System

Kupffer cells and hepatocytes, present in the liver, quickly remove the LPS from the blood [174], using the same method as intestine where endotoxins are also removed as liver is the first organ which receives blood from this organ. When LPS was injected into rats, its concentration was high, and it started to accumulate in the bile, which was then released into the gut. Somehow, these LPS‐induced TNF‐α levels, which are hepatobiliary factors, cause toxic effects on the intestine, which are then abolished by the bile [175]. However, during intraportal injection, Kupffer cells do not play any part in the activity, and hepatocytes are mainly involved in the removal of LPS. Very few information on Kupffer cell endotoxin removal are available [176].

#### 2.4.7 Concentration‐Dependent Activity

Biological activity of LPS depends on the concentration of CD14, BPI, LBP, cortisol, and MD‐2, and these can increase as well as decrease their activity. Transfer of LPS monomer to the CD14 can be carried out by LBP at low concentrations as well as at very high concentrations of LPS; LBP promotes its removal via non‐activating cellular pathway. However, some proteins, such as c‐reactive protein and mannose‐binding lectin, LBP, can bind with the LPS to avoid directly activating the cells [160].

#### 2.4.8 Chylomicrons

It was noted that for the removal of toxins from the microbes, chylomicrons and LBP play a vital role. Once it binds to LPS, it prevents cytokine production and starts to promote their removal from blood circulation. Production of these complexes in the gut shows a natural defense system against LPS [177].

#### 2.4.9 Internalization

LPS can be internalized due to the ingestion via alternative mechanism pathway, Fc receptor–mediated endocytosis of pathogen antibodies [178]. Fc receptor–mediated phagocytosis are the first line of defense that depends upon the CDC43 and RAC, whereas another mechanism is Rho dependent that is complement mediated phagocytosis [179].

#### 2.4.10 Cybernetics

For the removal of LPS, several systems are involved, such as reticulo‐endothelial organs named spleen, lymph nodes, and liver, that interact with the blood and the CNS to produce immune response. Signals between neuronal, endocrine, and immune systems show primarily mediated via the production of soluble immune and neuroendocrine mediators, which are testosterone, estrogen, ACTH, corticosteroids, some growth hormones, gonadotropin‐releasing hormones, leptin, ghrelin, prolactin, IGF‐1, endorphins, enkephalins as well as SNS (sympathetic nervous system) [159].

### 2.5 Integrated Strategy for the Separation of Endotoxin From Biofluids: LPS Capture on Newly Synthesized Protein

In this work, the authors used a blood cleaning procedure for the extracorporeal endotoxin separation via micro‐magnetophoretic devices. In this procedure, the first stage aims to LPS entrapment on functionalized magnetic nanoparticles, followed by the removal of loaded particles from biofluid in a second stage (Figure 9).

**FIGURE 9:**
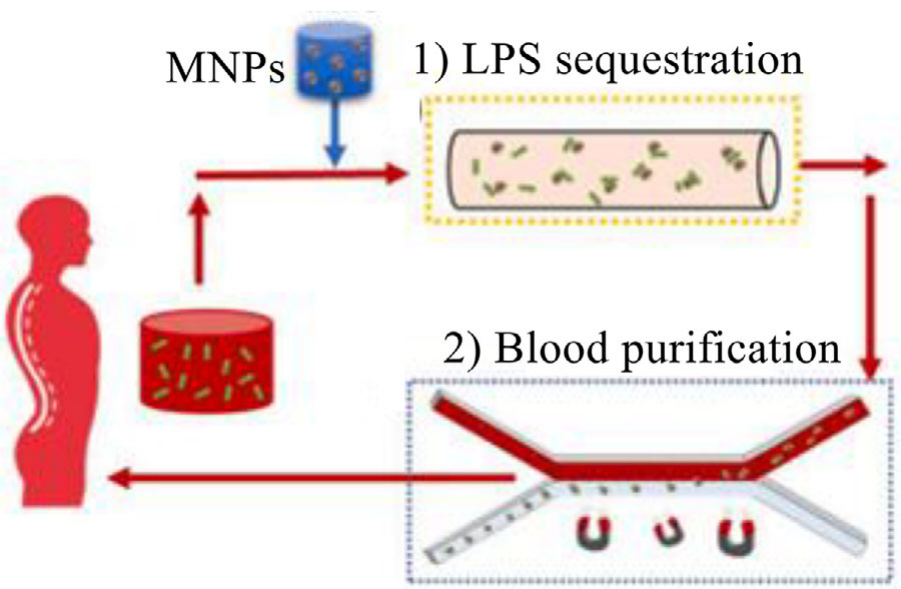
Schematic illustration of micro‐magnetophoretic extracorporeal blood cleansing process for LPS separation.

### 2.6 Blood Filtration and Endotoxin–Adsorption Strategy

To treat bacterial infection or endotoxemia, many studies are focusing on the endotoxin adsorption. In a previous investigation, scientists used loaded polymyxin B (PMB) onto gellan–polylysine polyion complex, and the obtained material was used to prepare wet‐spun fibers. The polymyxin‐loaded PLGP fibers could be easily knitted into the fabrics. The knit, dried, and rewetted fabrics from the yarns are shown in Figure 10A–D, respectively.

**FIGURE 10:**
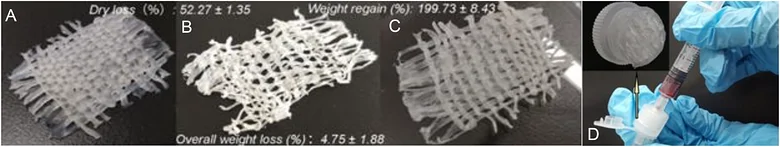
(A) As‐knitted, (B) dried, (C) rewetted, and (D) state of the PLGP fabric; illustration of PLGP fabric usage in hemoperfusion fiber.

The PMB content in the fiber was set at 10 mg/kg (in dry weight), and the formed yarns were strong enough for hemoperfusion. The fibers were biocompatible, and their endotoxin adsorption abilities were sufficient for endotoxin elimination in patients with endotoxemia. The adsorption amounts of the fibers in the SBF (simulated body fluid) and mouse plasma were 2.784 and 2.452 EU/mg, respectively. As reported by Hesse et al., the amount of endotoxemia caused by *E. coli*, which produces endotoxins in humans, is 20 EU/kg (by body weight). Therefore, the adsorption ability of these PLGP fibers was enough for patients with endotoxemia.

### 2.7 LPS‐Neutralization as a Therapeutic Target

Sepsis is a fatal disease resulting from bacterial or fungal infections. Because endotoxin (LPS) is a major pathogenic factor in sepsis caused by Gram‐negative bacterial infections, the neutralization of LPS is a promising therapeutic target. To effectively eliminate LPS and to protect infected mice from endotoxic shock, Jiang et al. [180] developed a hybrid macrophage mimetic liposome (called M‐Lipo) capable of neutralizing and eliminating LPS (Figure 11).

**FIGURE 11:**
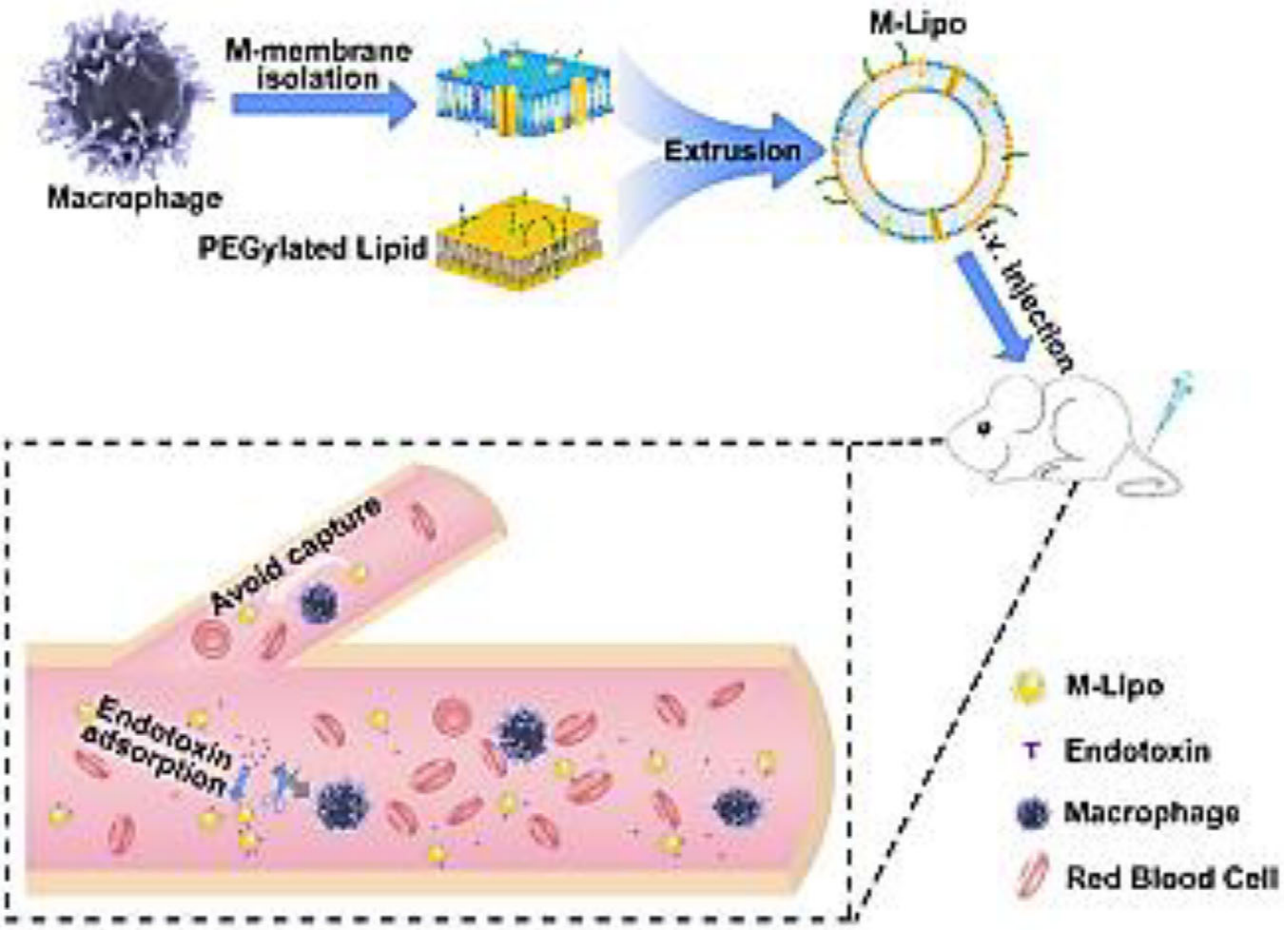
Schematic illustration of M‐Lipo preparation and application for sepsis.

Fusing a macrophage membrane with artificial PEGylated lipids developed this M‐Lipo. The results showed that M‐Lipo had surface proteins like those of macrophages and that it could adsorb LPS substantially. Thanks to the PEGylated lipids, M‐Lipo had much better stability and prolonged blood circulation. In addition, pharmacokinetic results revealed that M‐Lipo clearly had a longer retention (16%) in the blood (at 12 h) than the natural macrophage membrane, called M‐membrane (3.3%). The results obtained showed that the M‐Lipo hybrid not only reduced the toxicity of LPS in vitro but also protected the mouse from endotoxic shock in vivo (Figure 12).

**FIGURE 12:**
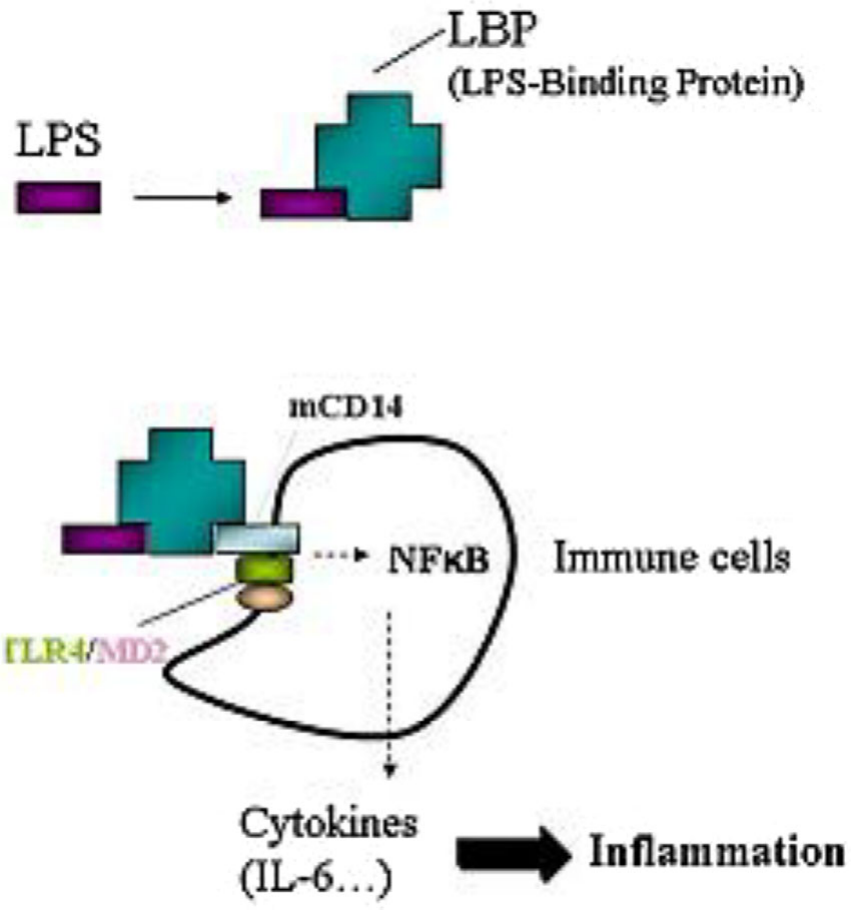
Proinflammatory cascade induced by LPS. IL6, interleukin‐6 (inflammatory cytokine); LBP, lipopolysaccharide‐binding protein; LPS, lipopolysaccharide; mCD14, membrane cluster of differentiation 14; MD2, myeloid differentiation protein‐2; NF‐κB, nuclear factor κB; TLR4, toll‐like receptor 4.

### 2.8 Proinflammation Mechanism Induced by Endotoxin (LPS)

### 2.9 Biological Effects of Bacterial Lipopolysaccharide (Endotoxin) in Fish

Endotoxin, a component with properties, plays a vital role in the innate immune response of different eukaryotic species, including fish [63]. Its conserved structural sequences can have effects on the system by influencing various types of immune cells such as T cells, B cells, and white blood cells/lymphocytes. In fish, endotoxin actively stimulates the growth of B‐lymphocytes, leading to the activation of genes like IL‐1 when fish white blood cells are exposed to LPS. Moreover, endotoxin acts as a stimulant for antibody production in fish. It can directly stimulate B cells [64] and trigger antibody‐mediated immune reactions. Additionally, endotoxin enhances the components and survivability of fish when administered in high amounts. However, the immunomodulatory activity of LPS may be affected by its ability to disperse in water. Stress conditions in fish can cause changes in their metabolic responses, including increased cortisol levels that are often triggered by exposure to endotoxin. Future research should focus on investigating the toxicity and effects of LPS in fish species while considering their habitats, strains, and physical characteristics of LPS that greatly affect its biological activities [65]. This research plays a role in comprehending the biological impact of endotoxins and their potential consequences on the health of both humans and animals.

### 2.10 Extraction and Determination of Endotoxins in Oil

A recent study has shown that it is possible to measure the levels of endotoxins in water extracts of pure paraffin oil using the gel clot method [181]. The study establishes a validated limit value of 0.25 EU/mL, which can be considered an analytical approach for medical devices that contain oily components and are not easily soluble in water [182]. This method expands the range of endotoxin analysis to encompass devices, thereby improving the overall comprehensiveness and usefulness of endotoxin testing for quality control and safety assessment purposes in the field of medical devices [183]. In order to confirm that the aqueous phase extracted from paraffin oil does not interfere with clot formation between Tachypleus Amebocyte Lysate and endotoxins, an interference test was conducted [184]. Various concentrations of endotoxins were prepared in paraffin oil. Their recovery was tested using a dynamic turbidimetric method. All results were observed within the range of 86.8%–96.8%, meeting the requirement set by Pharmacopeia (50%–200%). This also demonstrates that the test method utilized in this study accurately determines the concentration of endotoxins in paraffin oil by analyzing its aqueous phase extract [185].

Recent advances in endotoxin research highlight both progress and persistent challenges. Although the LAL assay remains the gold standard for detection, it faces limitations such as low sensitivity in complex matrices and ethical concerns over horseshoe crab use. Newer approaches, including recombinant factor C assays and biosensor‐based technologies, show promise but lack widespread standardization. Beyond methodological issues, significant knowledge gaps remain: Little is known about the health impacts of chronic low‐level exposure, synergistic effects with environmental pollutants, and population‐specific vulnerabilities. Moreover, comparative studies across species, age groups, and genders are scarce, limiting the generalizability of findings. Addressing these gaps is essential not only for refining laboratory and occupational safety standards but also for strengthening public health responses to environmental and clinical endotoxin exposure.

#### 2.10.1 State of the Art

It was noted that commercially available food, protein, and biologically active substances have a higher number of endotoxins, whereas their presence, even in small amounts in recombinant protein preparation, could produce some serious side effects in host body, such as shock, tissue injury, and sometimes death. To avoid this, endotoxin has to be removed from drugs, biological, and pharmaceutical products. Their adverse effects are known, but standardization and preventive measurements are also needed immediately. Endotoxins that are exposed by the environment can produce inflammation response in early life, whereas continuous exposure can produce immunity against them in healthy individuals. In recent years, two‐phase aqueous micellar systems were introduced, but more research is required in that case as well [186].

Inflammatory response is produced when cytokines, ILs, prostaglandins, and TNF factors are produced due to the presence of endotoxins. Aside from the inflammation, they also produce some shock response, whereas C3a and C5a produce histamine, which is a major effector for the allergens and causes vasodilation and inflammation. Anti‐endotoxin therapy cannot achieve significant improved outcomes yet in human with sepsis. More studies are required in this aspect, whereas scientists express more modesty regarding the understanding of the endotoxin roles in the septic shocks.

However, it is essential to maintain these LPSs at minimum level to avoid any mishaps in various manufacturing categories, like pharmaceuticals, food such as seafood, medical devices, and waste‐dumping industries. On the other hand, LAL assay is considered the best detection method for the LPS; however, it is necessary to develop more methods that are more accurate, fast, and reliable, similar to optical detection such as fluorescence that is very sensitive. A great disadvantage of this method is the degradation of the fluorophore or possible quenching effects [187]. However, the removal of circulating LPS from bloodstream can be carried out by hemoperfusion method using Polymyxin B cartridge. Results are significant, in which CD64 monocytes and others reduce mortality to its normal range of 33%. More research is required in this aspect [157, 186].

LPS could enter the body through the use of medical devices, such as urinary catheters, and lead to UTI that is due to the formation of biofilms within the surface of these devices [188]. Similar medical devices cause the entry of LPS at different levels, such as other catheters, as well as syringes, IV sets, blood bags, and urine bags. It is important to minimize their exposure to the body, which can be possible if medical devices are properly sterilized following terminal sterilization methods with high‐level disinfectant such as H_2_O_2_ gas [189] or ethylene oxide gas with proper concentration, humidity, and pressure. Medical devices should be correctly tested using LAL method, whereas some new methods should be developed to monitor the sterility at a fast rate. Biological indicators are also a source to make a decision of product sterility as they are not authentic and sometimes can produce some false results. Similar to these sterilization methods, gamma rays, E‐beam, and dry heat sterilization methods are also followed due to their long cycles and required special facilities; therefore, they should be discontinued. For the sterilization of medical devices with ETO or H_2_O_2_, workers should be trained to avoid compromised sterility. Similar to wastewater and biological material, membrane filters and anti‐LPS proteins, respectively, are used for its removal, whereas peptide‐based biosensors, aptamer‐based biosensors, and antibodies as well as cell‐based biosensors can be used for its detection. However, more research should be carried on in this aspect. Similar to these biosensors, some nanomaterial biosensors are also used. An important issue is how to remove endotoxins in an economical way. This issue is not solved satisfactorily, and more research is required in this field to remove or destroy these endotoxins in an economic way.

### 2.11 Established and Emerging Methods

Endotoxin detection is essential in pharmaceuticals and medical devices. Traditional methods include the Rabbit Pyrogen Test (RPT), approved by the FDA in the 1920s but limited by high cost and long testing times [71, 184]. The LAL assay, based on clotting of horseshoe crab blood in response to LPS, is now widely used in parenteral drug and device testing. It has evolved into gel clot, chromogenic, and turbidimetric formats but is sensitive to protease interference and β‐(1,3)‐d‐glucan contamination [184].

To overcome such limitations, biosensor technologies are being developed. Protein‐based sensors (e.g., CD14, LBP, BPI, CAP18) and peptide‐ or antibody‐based sensors provide specificity but face challenges of cost, complexity, and labor [185, 190, 191]. Aptamer‐based biosensors offer stability and high specificity, whereas cell‐based sensors use macrophages or other immune cells as recognition elements. Nanomaterials, such as gold nanoparticles, polymers, and molecularly imprinted polymers, are also being applied to enhance electrochemical detection. Although promising, these approaches are not yet standardized for routine use. Current research is focused on synthetic recognition elements, miniaturization, and biochip platforms, with progress requiring integration of chemistry, biology, materials science, and engineering to deliver reliable and cost‐effective endotoxin detection [192].

## 3 Conclusion

This review has examined the different routes of endotoxin exposure through blood, skin, inhalation, and contaminated medical devices, and the approaches used to detect, neutralize, and remove them. The evidence shows that endotoxins represent serious risks for both human and animal health, from acute inflammation to long‐term metabolic disorders, with particular concern in occupational and environmental settings such as wastewater treatment and aquaculture.

Looking ahead, there is a clear need for faster, more reliable, and standardized detection tools, as well as better strategies for neutralization. Strengthening sterilization standards, monitoring environmental contamination, and investigating the effects of chronic low‐level exposure should be public health priorities. Future research should focus on affordable biosensors, species‐specific responses, and long‐term health outcomes to better protect both clinical safety and environmental health.

## Author Contributions

**Uzair Ishtiaq**: conceptualization, methodology, software, data curation, investigation, validation, formal analysis, supervision, funding acquisition, visualization, project administration, resources, writing – original draft. **Nicolas Joly:** methodology, investigation, formal analysis, validation. **Vincent Lequart:** project administration, resources, formal analysis. **Patrick Martin**: conceptualization, methodology, software, data curation, investigation, validation, formal analysis, supervision, funding acquisition, visualization, project administration, resources, writing – original draft, writing – review and editing. **Marielba Morillo:** conceptualization, methodology, validation, visualization, writing – review and editing. **Janne Rojas**: conceptualization, methodology, investigation, validation, supervision, visualization, writing – original draft, writing – review and editing.

## Ethics Statement

The authors have nothing to report.

## Conflicts of Interest

The authors declare no conflicts of interest.

## Data Availability

The data that support the findings of this study are available on request from the corresponding author. The data are not publicly available due to privacy or ethical restrictions.
